# Public health round-up

**DOI:** 10.2471/BLT.22.010522

**Published:** 2022-05-01

**Billions breathing unhealthy air**

Air pollution in a fishing village in Lagos, Nigeria where vehicle emissions, diesel generators and the burning of biomass impact the community’s health. According to the latest update of the World Health Organization’s (WHO) air quality database, an estimated nine out of ten assessed villages, towns and cities are exposed to air that exceeds WHO evidence-based recommendations for particulate matter concentrations, while close to eight out of ten are exposed to unsafe levels of nitrogen dioxide.

**Figure:**
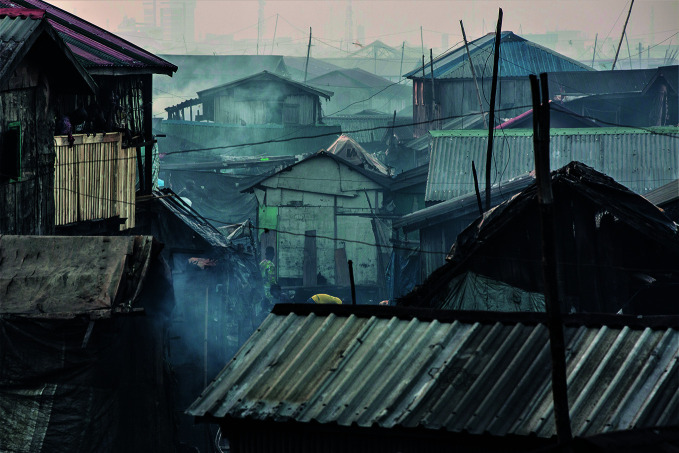
UNICEF/Tanya Bindra

## Attacks on health care in Ukraine

More than 100 attacks on health care have taken place in Ukraine since the war began on 24 February. A total of 103 attacks had been reported by 7 April, including 89 on health facilities and 13 on health-care transport, including ambulances. The attacks had claimed 73 lives and injured 51 people.

In a 7 April statement, WHO Director-General Tedros Adhanom Ghebreyesus once again called on the Russian Federation to stop the war. “We are outraged that attacks on health care are continuing,” he said, pointing out that attacks are a violation of international humanitarian law.

https://bit.ly/3KpW3zw

## Delivering HIV treatment in Ukraine

Working in collaboration with the Ukrainian authorities, WHO and partners ensured the shipment of human immunodeficiency virus (HIV) antiretrovirals to cover the treatment needs of Ukrainian people for the next 12 months.

Responding to reports of HIV treatments being disrupted by the war in Ukraine, WHO, together with the United States President’s Emergency Plan for AIDS Relief, the Global Fund to Fight AIDS, Tuberculosis and Malaria, the Ukrainian Ministry of Health’s Public Health Centre, and the nongovernmental organizations Alliance for Public Health and 100% Life, procured 209 000 packs of the antiretrovirals tenofovir, lamivudine and dolutegravir. The first shipment was delivered at the beginning of March.

According to a 6 April report, as of 21 March, 36 out of 403 antiretroviral treatment sites in the country were closed, while most of those still open were either fully or partially functioning.

An estimated 260 000 people are living with HIV in Ukraine. Prior to the war, over half, or nearly 150 000 people, were on antiretroviral treatment, including more than 2700 children.

https://bit.ly/3KpQbq5

## Efficacy of single-dose human papillomavirus vaccination

A single-dose human papillomavirus (HPV) vaccine protects against HPV, a virus that can cause cervical cancer. This is a key conclusion of the WHO Strategic Advisory Group of Experts on Immunization which has been evaluating emerging evidence regarding the efficacy of single-dose regimens relative to two- or three-dose regimens, and which met in April to discuss its finding.

Single-dose regimens could improve prevention of the disease, being less costly, less resource-intensive and easier to administer. Single-dose regimens also facilitate catch-up campaigns for multiple age groups, and reduce the challenges linked to tracing recipients for a second dose.

https://bit.ly/3KqaRhL

## Supply of Covaxin suspended

WHO suspended the supply of coronavirus disease 2019 (COVID-19) vaccine Covaxin through United Nations procurement agencies.

Confirmed on 2 April, the suspension was a response to recently identified deficiencies in good manufacturing practices. Bharat Biotech, the vaccine manufacturer, committed to addressing the deficiencies and is developing an action plan for submission to the Drugs Controller General of India and WHO.

In the interim, and as a precautionary measure, the company has indicated that it will suspend production of Covaxin for export. Supply of the vaccine will be interrupted for the foreseeable future.

https://bit.ly/3uqXsk2

## Extensively drug-resistant Shigella in Europe

The United Kingdom of Great Britain and Northern Ireland is investigating a cluster of 84 cases of extensively drug-resistant (XDR) *Shigella sonnei* which were reported between 4 September 2021 and 1 March 2022. This compares with 16 cases, none of which were XDR, reported in a 17-month period between 1 April 2020 and 31 August 2021. According to a 24 March report, the people infected are widely distributed across the United Kingdom. Infections have also been reported in other European countries.

Although most *S. sonnei* infections result in relatively mild disease (typically diarrhoea, fever and stomach cramps) with symptoms lasting around seven days, multidrug-resistant and XDR shigellosis are of concern because treatment options are very limited for moderate to severe cases.

National health authorities are conducting epidemiological and genomic investigations to determine the route of transmission and links with the strain detected in the United Kingdom. WHO has requested that national authorities report cases of drug-resistant *S. sonnei* through the Global Antimicrobial Resistance and Use Surveillance System and share information with the relevant health services.

https://bit.ly/3LSkZQu

## Yellow fever in Kenya

The Ministry of Health of Kenya declared an outbreak of yellow fever on 4 March. As of 15 March, a total of 53 people were reported to have been infected, six of whom died of the disease (case fatality ratio: 11.3%).

The outbreak is centred on Isiolo county in central Kenya, roughly 270 km north of the capital, Nairobi. Despite being in a remote pastoralist (livestock herding) area and sharing no international borders, Isiolo county is marked by frequent population movements (recently increased by an ongoing drought which is driving the movement of pastoralists from other areas), an influx of refugees from neighbouring Somalia and informal mining activities which have attracted large numbers of workers. The presence of non-human primates in the area is also judged to increase the risk of spread to other areas.

In view of these factors, WHO assesses the risk of spread to be high at the national and regional level and encourages Member States to take all actions necessary to keep travellers informed of risks and preventive measures including vaccination.

https://bit.ly/3urdSZt

## Technology transfer to Indian manufacturer

WHO’s Product Development for Vaccines Advisory Committee selected Indian manufacturer BiologicalE (Bio E) to be the latest recipient of mRNA technology from the WHO technology transfer hub. Bio E already manufactures a number of vaccines, including Corbevax, a second-generation vaccine for COVID-19.

According to the 31 March announcement, WHO and partners will work with the Indian government and Bio E to develop a roadmap and put in place the training and support needed for the company to start producing mRNA vaccines as soon as possible.

Launched in June 2021 and located in Cape Town, South Africa, the objective of the WHO technology transfer hub is to build capacity in low- and middle-income countries to produce mRNA vaccines. However, the hub has the potential to support the manufacture of other products and target other priorities including, for example, malaria, HIV and cancer.

https://bit.ly/3LSLBAV

**Cover photo**

People displaced by conflict in the Tigray region of Ethiopia use donkeys to transport water. For several months, the population has been suffering from the drought affecting the region of Afar in the north of the country.

**Figure:**
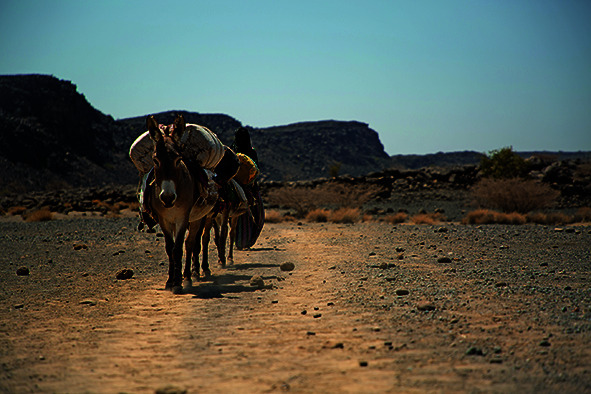
UNICEF/Pouget

## New air quality data

An estimated nine out of ten assessed settlements (ranging from fewer than 100 to more than 30 million inhabitants) are exposed to air that exceeds WHO recommended average annual limits of 15 micrograms per cubic metre of air for particulate matter with a diameter equal to or less than 10 μm (PM_10_) and five micrograms per cubic metre of air for particulate matter with a diameter equal to or less than 2.5 μm (PM_2.5_). An estimated eight out of 10 are exposed to air that exceeds the WHO recommended average annual limits of 10 micrograms per cubic meter of nitrogen dioxide (NO_2_).

This is according to the 2022 update of WHO’s ambient air quality database which was released on 4 April in the lead-up to World Health Day, which this year celebrated the theme “Our planet, our health.” The findings have prompted WHO to highlight the importance of curbing fossil fuel use and taking other tangible steps to reduce air pollution levels.

The evidence base for the harm caused by air pollution has been growing rapidly and points to significant harm caused by even low levels of many air pollutants. Last year, WHO responded by revising its Air Quality Guidelines to reflect the evidence, making them more stringent, especially for PM and NO_2_, a move strongly supported by the health community, medical associations and patient organizations.

https://bit.ly/3xk6zEL

**Looking ahead**

3–5 May, Geneva Health Forum. https://bit.ly/3HJ5wQt

22–28 May, Seventy-fifth World Health Assembly. https://bit.ly/3IK55GL

7–10 June, World Hepatitis Summit. https://www.worldhepatitissummit.org/

