# Corrigendum

**DOI:** 10.2471/BLT.22.101222

**Published:** 2022-12-01

In: Public health round-up. Bull World Health Organ. 2022 May 1; 100(5):296–297, on page 297, middle column, the second sentence should read as follows: 

“As of 15 March, a total of 53 people were reported with suspected Yellow fever infections, six of whom died of the disease (case fatality ratio: 11.3%).”

Public health round-up. Bull World Health Organ. 2022 May 1;100(5):296–297. https://dx.doi.org/10.2471/BLT.22.010522

